# Promoting integrated care in prostate cancer through online prostate cancer-specific holistic needs assessment: a feasibility study in primary care

**DOI:** 10.1007/s00520-019-04967-y

**Published:** 2019-07-23

**Authors:** Amy L. Clarke, Julia Roscoe, Rebecca Appleton, Deepak Parashar, Radha Muthuswamy, Omar Khan, Jeremy Dale, Veronica Nanton

**Affiliations:** 1 University of Warwick, Coventry, UK; 2 Statistics and Epidemiology Unit, Warwick Medical School, University of Warwick, Coventry, UK; 3 The Alan Turing Institute, London, UK; 4 Warwick Cancer Research Centre, University of Warwick, Coventry, UK; 5 Prostate Cancer UK Information Technology Consultant, London, UK

**Keywords:** Holistic needs assessment, Survivorship, Primary care, Cancer follow-up, Digital health

## Abstract

**Purpose:**

This study assessed the feasibility of implementing a novel model of integrated prostate cancer care involving an online prostate cancer-specific holistic needs assessment (sHNA) and shared digital communication between patients and their healthcare professionals (HCPs). The sHNA produces a semi-automated care plan that is finalised in consultation between the patient and their practice nurse.

**Methods:**

Men living with and beyond prostate cancer were invited to participate in a 9-month non-randomised cluster controlled feasibility study. The intervention group was asked to complete the sHNA on three occasions. Data were collected using Patient Reported Outcome Measures (PROMs) at baseline, 10 and 24 weeks, and 9 months. Outcomes included recruitment, retention, acceptability, and engagement with the sHNA and PROMs.

**Results:**

Fourteen general practices (8 intervention and 6 control), and 41 men (29 intervention and 12 control) participated. Initial patient engagement with the sHNA was high, with all but one receiving practice nurse-led follow-up and an individualised care plan. The sHNA proved useful in identifying ‘red flag’ symptoms, and helping practice nurses decide when to seek further medical care for the patients. There was a high level of acceptability for patients and HCPs. However, integration of care did not occur as intended because of problems linking hospital and general practice IT systems.

**Conclusion:**

While the study demonstrated the feasibility of implementing the sHNA, it did not meet the a priori progression criteria; as such, undertaking a definitive randomised controlled trial is not appropriate until the identified methodological and technical issues have been addressed.

## Introduction

Prostate cancer is the most common cancer in men living in the UK, with over 47,000 cases diagnosed annually [1]. Ten-year survival rates are high (84%), but cancer survivorship is placing an increasing demand on already stretched specialist services [1]. Consequently, many men living with or beyond prostate cancer report on-going needs, with concerns around urinary, bowel and sexual dysfunction, as well as general information [2]. Patient-perceived unmet needs have been shown to be associated with decreased quality of life in cancer survivors [3]. Changes to the organisation of services are required to ensure needs are more effectively addressed.

Primary care has traditionally had limited involvement in comprehensive cancer care [4]. However, patients who are stable or whose cancer has been successfully treated are increasingly being offered primary care follow-up in line with recommendations from the National Institute of Care Excellence [5]. Guidance on follow-up for these patients, however, has been very limited.

Integrated models of care have been proposed as a way of optimising the treatment of cancer and follow-up care [6]. However, primary and secondary care services are often fragmented; with poor communication, lack of coordination, and role definition indicated as persistent problems [7]. Furthermore, patients living with and beyond cancer often report a desire to be actively involved in their care, but many feel excluded from care planning and shared decision making [8].

Holistic needs assessments (HNA) are recommended in the treatment and follow-up care for cancer and aim to provide a mechanism to help patients more easily identify and disclose their needs to healthcare professionals (HCPs), and aid the development of person-centred care plans. The sharing of care plans has been identified as a facilitator to improve communication and care coordination [8]. However, HNA are not routinely carried out during follow-up for men with prostate cancer.

Digital health technology has the potential to better enable an integrated model of care via improvements in communication and information transfer [9]. When applied to the context of HNA, digital health technology could facilitate the sharing of identified needs and care plans, between services and with the patient.

We developed an online prostate cancer-specific holistic needs assessment (sHNA) [10] with the aim of promoting an integrated approach to care through enhanced communication between the patient and health care providers in specialist and generalist settings. The sHNA includes 11 domains that encompass prostate cancer symptoms and a range of broader issues. The system is adaptive, enabling patients to choose domains for self-assessment that are deemed personally relevant, and it also encompasses external links to curated sources of information in response to specified concerns. In this way, it is intended to support men in identifying and communicating their on-going needs to their HCPs and to facilitate self-management. It also allows the online sharing of care plans between HCPs and the patient, thus promoting an integrated approach to care through enhanced communication.

The aim of the current study was to test the feasibility of undertaking a primary care-based randomized cluster controlled trial in terms of recruitment, retention, engagement, and acceptability of the intervention. This paper addresses implementation (extent to which the intervention was delivered as intended), practicality (extent to which the intervention was carried out with intended participants), and acceptability (extent to which the intervention is deemed attractive to those delivering it or programme recipients) [11].

## Methods

### Study design

This study is a non-randomised clustered controlled trial involving two groups (intervention and control); the full protocol has been published previously [10]. Prior to study commencement and due to delays in site opening, a substantial amendment was approved to reduce the study duration from 12 to 9 months.

### Participants

General practices (primary health care providers) were recruited in one area of the West Midlands, United Kingdom. Practice eligibility was determined by a referral pathway to a single specialist centre. Intervention sites were required to support a practice nurse to partake in a training programme run by Macmillan Cancer Support. This programme formed part of a national roll out. The 5-day course included an introduction to the biology and epidemiology of cancer, cancer staging and grading, treatment types and treatment consequences, impact on work, finances and relationships, communication skills, and support for the cancer patient. Nurses who had previously undertaken the training were also eligible to run the study. The study team provided an additional 2-day prostate cancer specific programme held at the specialist centre for all the participating nurses.

Men were eligible to take part if they had ever received a diagnosis of prostate cancer or treatment from the participating specialist service, were able to read, understand and communicate in English, and were able to provide informed written consent. Men were excluded if they did not meet the criteria, suffered from severe mental health problems, were unable or unwilling to complete outcome assessments, and were living in a care setting.

Men with prostate cancer were identified from practice lists by practice administrators and screened for eligibility by their general practitioner (GP). Eligible patients were sent postal invitations along with relevant patient information materials to consider, and were asked to return the expression of interest reply slip. Eligible patients were also invited to take part on an opportunistic basis during routine appointments. Written consent from all participants was taken by a member of the study team.

### Ethics

This study was approved by the East Midlands - Nottingham 2 Research Ethics Committee (REC reference: 15/EM/0534).

### Procedures

#### Control

Participants at control practices received usual care which was either provided by the specialist centre or by the general practice team depending on the individuals care pathway.

#### Intervention

Participants at intervention practices were offered the online sHNA and practice nurse follow-up, described in detail elsewhere [10]. They were invited to complete the sHNA at three-time points (baseline, 3 months, and 6 months). Following each completion, men were invited to make an appointment with their nurse to discuss any concerns raised during the assessment and to complete their personalised care plan. Appointments were undertaken by telephone or face-to-face at the patient’s practice. An indication of any serious or life-threatening physical or psychological concerns produced a ‘red flag’ pop up advising the patient to contact their GP team urgently.

### Outcome measures

#### Feasibility and acceptability

A priori criteria for study feasibility and acceptability were as follows:

- Recruitment: consent rate of ≥ 25% of eligible men patients approached about the study
- Engagement and follow-up data feasibility and acceptability: ≥ 70% completion of the sHNA and outcome measure by the intervention group at each time point.
- Intervention acceptability and usability were assessed using an adapted version of a Technology Acceptance Model (TAM) questionnaire and qualitative interviews.

#### Patient-reported outcome measures

PROMs were completed either online or on paper and returned via the post and included Health-related Quality of Life [The EuroQol five dimensions questionnaire (EQ5D)] [12], Cancer Specific Quality of Life [European Organisation for Research and Treatment of Cancer Quality of Life Questionnaire (EORTC-QLQ)] [13], Prostate Cancer Symptoms [Expanded Prostate Cancer Index Composite], Cancer Survivor Unmet Needs [Cancer Survivors Unmet Needs instrument] [14], mental well-being [The Warwick and Edinburgh Mental Well-being Scale] [15], and Patient Activation [Patient Activation Measure] [16]. Intervention participants were also invited to complete an adapted version of the Technology Assessment Model (TAM) at T4. All patient participants were asked to complete PROMs at baseline (T1) and 9 months (T4). Interim assessments using a sub-set of PROMs (Cancer Survivor Unmet Needs and Patient Activation Measure) were conducted with the intervention group at 10 weeks (T2) and 24 weeks (T3).

#### Sample size

As this was a feasibility study, no formal sample size calculation was undertaken. However, the sample size was determined on the basis of practice list sizes (range < 2000 to > 10,000) in the participating area with direct referral pathways to the specialist site. It was estimated from population data that a mean list size of 5000 patients would generate 30–40 men with a diagnosis of prostate cancer. Thus, from our anticipated ten intervention and four control practices, we predicted that approximately 283 would fulfil eligibility, and that 85 men (~ 30%) would consent. With an anticipated attrition rate of 20%, we estimated study completion by approximately 68 patients, 49 intervention, and 19 control participants when allocated to the available sites. An original sample size of 200 patients (150 intervention and 50 control participants) was superseded for the calculation above after list sizes were found to be substantially smaller than the anticipated ~ 75 eligible patients per practice.

### Statistical analysis

Descriptive statistics were used to summarise baseline characteristic data. Continuous variables were summarised as median [interquartile range (IQR)] and percentages were used for categorical variables. Median change from baseline to post intervention (at least 9 months) are reported for PROMs. Missing data were handled using the SPSS default listwise for each instrument. Significance testing was not carried out owing to the fact that the study was not powered to detect changes on any of the measures, low numbers, and high levels of missing data.

The adapted TAM was summarised using frequencies, and web analytics were used to determine the percentage of patients engaging with each of the domains of the sHNA.

## Results

### Recruitment and retention

**Fig. 1:**
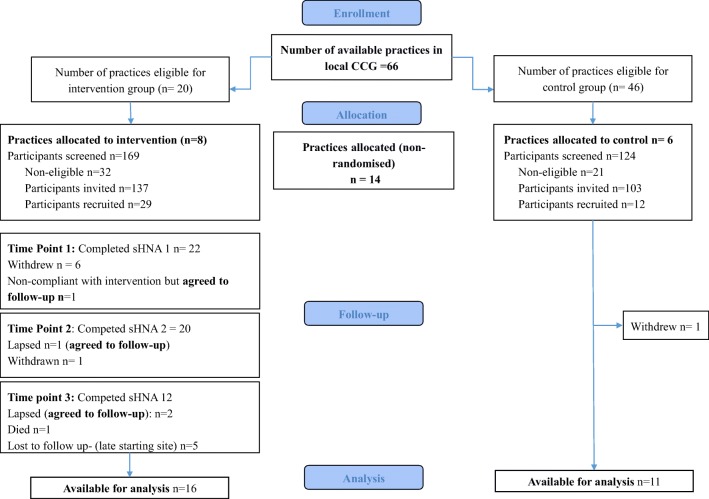
Consort diagram to show participant flow through study

The flow of participants is highlighted in Fig. 1.

Recruitment of the 14 participating general practices (8 intervention and 6 control) took place between February and September 2017. Five nurses were recruited to the study and undertook additional prostate cancer training. One nurse withdrew prior to implementation. Of the remaining four, two were regular practice nurses, and two were practice nurses with a dual research role, asked to deliver the intervention at their own practice and others. Overall, 240 men were invited to participate, with 41 (17%) subsequently recruited; 29 (21%) were at intervention practices and 12 (13%) at controls. Recruitment rates varied between practices from 10/12 eligible patients at one intervention group practice to 1/27 in one control practice. Of those allocated to the intervention group, 17 (59%) did not complete the study (Fig. 1); however, four of these participants did agree to complete follow-up assessments. Of those allocated to the control group, 1 (8%) did not complete.

Patient baseline characteristics, stratified by intervention group, are shown in Table 1. Groups were generally well-matched; however, the intervention group had a longer duration since diagnosis (65 vs 59 months) and a greater proportion of participants over the age of 71 (76% vs 59%).

**Table 1:** Patient baseline demographics stratified by group

	Intervention (*n* = 29)	Control *n* = 12	Total (*N* = 41)
Time since primary diagnosis (months)	65 (48–129)	59 (50.25–120.5)	63 (49–127.5)
Age (*n*, %) years			
45–50	0 (0)	0 (0)	0 (0)
51–55	1 (3)	1 (8)	2 (5)
56–60	0 (0)	0 (0)	0 (0)
61–65	4 (14)	1 (8)	5 (12)
66–70	2 (7)	3 (25)	5 (12)
71–75	8 (28)	1 (8)	9 (22)
76–80	7 (24)	2 (17)	9 (22)
81–58	7 (24)	2 (17)	9 (22)
85+	0 (0)	2 (17)	2 (5)
Primary treatment (*n*, %)			
Radical prostatectomy	10 (34)	4 (33)	14 (34)
Radiotherapy	8 (28)	0 (0)	8 (20)
Active surveillance	5 (17)	2 (17)	7 (17)
Hormones	4 (14)	1 (8)	5 (12)
Transurethral section of the prostate	1 (3)	1 (8)	2 (5)
Cystoprostatectomy	1 (3)	0 (0)	1 (2)
High intensity focal ultrasound	0 (0)	1 (8)	1 (2)
Unknown	0 (0)	3 (25)	3 (7)
Ethnicity (*n*, %)			
White British	22 (76)	6 (50)	28 (68)
White Irish	2 (7)	0 (0)	2 (5)
Asian or Asian British Pakistani	1 (3)	0 (0)	1 (2)
Black or Black British African	0 (0)	1 (8)	1 (2)
Black or Black British Caribbean	2 (7)	0 (0)	2 (5)
Missing	2 (7)	5 (42)	7 (17)
Comorbidities (*n*, %)			
Diabetes	7 (24)	3 (25)	10 (24)
CHD	3 (10)	1 (8)	4 (10)
Arthritis	6 (21)	4 (33)	10 (24)
COPD	4 (14)	2 (17)	6 (15)
CKD	4 (14)	3 (25)	7 (17)
Hypertension	8 (28)	4 (33)	12 (29)
Other cancer	5 (17)	1 (8)	6 (15)
Other	17 (59)	2 (17)	19 (46)
At least one comorbidity	23 (79)	10 (83)	33 (81)
Total number of comorbidities	2.5 (1–4.00)	1 (1–2.75)	2 (1–4)
Missing	1 (3)	0 (0)	1 (2.4)
Living arrangements (*n*, %)			
With partner	20 (69)	9 (75)	29 (70)
With relatives	1 (3)	0 (0)	1 (2)
Alone	7 (24)	3 (25)	10 (24)
Missing	1 (3)	0 (0)	1 (2)
Caring responsibility (*n*, %)	2 (7)	0 (0)	2 (5)
Missing	1 (3)	0(0)	1 (2)

Retention rates were higher in the control group, with participants in this group being less representative of men over the age of 71 years, and more representative of those closer to point of diagnosis when compared to the intervention group. Whilst this might look to indicate a relationship between age and time since diagnosis on retention, we deemed a between group analysis inappropriate based on the small number of study participants. However, for interest purposes only, we conducted further descriptive analyses to investigate age and time since diagnosis on the retention rates observed in the intervention group among those who had the opportunity to complete the study (i.e. excluding the participants from the late starting site). Among study completers, we observed no difference in terms of age between older (71–85+ years) and younger (45–70 years) participants with retention being 50% for both groups. Median time since diagnosis was longer among study completers (120 vs 60 months).

### Intervention implementation

Evidence of engagement and the implementation profile of the intervention group is shown in Table 2. Implementation of the intervention was relatively consistent with our implementation plan as described in the protocol [10]. Engagement was assessed by sHNA completion rates, of which all but one were followed-up with a nurse-led consultation and personalised care plan; reasons for this were due the patient’s admission to hospital. The most common areas of concern indicated by web analytics reviewing domain access included ‘emotional and psychological concerns’, ‘illness and treatment’, and ‘access and services’. Consultations were predominately telephone-based and lasted for a median of 15 min. As intended, the sHNA highlighted symptoms of serious concern ‘red flags’ to both the patient and their HCPs, and based on sHNA outputs and nurse consultation, practice nurses made seven referrals for men to receive more specialist care. Data on referrals were not available for the control group.

**Table 2:** Implementation profile for n = 29 participants allocated to the intervention group

	sHNA1	sHNA2	sHNA3
Participants completed HNA, *n* (%)	22 (76)	20 (69)	12 (41)
Participants receiving a nurse consultation (*n*)	21	20	12
Reasons for missed consultation			
Poor health/ in hospital (*n*)	1		
Consultation type			
Telephone (*n*)	19	16	12
Surgery (n)	2	4	0
Length of consultation minutes			
median (range)	15 (10–25)	15 (13–25)	15 (10–20)
Domains of sHNA accessed *n* (%)			
Physical	8(28)	5(17)	4(14)
Emotional	17(59)	16(55)	14(48)
Illness treatment	10(34)	9(31)	8(28)
Information, Communication	9(31)	9(31)	1(3)
Independence	8(28)	9(31)	1(3)
Finance	6(21)	8(28)	0(0)
Occupational	7(24)	8(28)	4(14)
Rehabilitation	6(21)	5(17)	2(7)
Access and services	13(45)	9(31)	1(3)
Religious	7(24)	6(21)	4(14)
Legal	5(17)	5(17)	3(10)
Number of ‘red flags’ recorded			
Physical	12	10	7
Emotional	0	1	0
Referrals *n* (%)			
None	19 (90)	14(70)	11 (92)
GP	1 (5)	2 (10)	1(8)
Continence team	1(5)	0 (0)	0 (0)
Clinical nurse specialist	0 (0)	2 (10)	0 (0)
Missing	0 (0)	2 (10	0(0)

Reasons for missed sHNA are detailed in Fig. 1

### Patient-reported outcome measures

Follow-up data were available for 16 intervention and 11 control participants. Table 3 displays completion rates at each time point. Descriptive data stratified by group are presented for PROMs completed at T1 and T4 (Table 4). Data for the intervention group did not appear to denote a clear positive change in the direction of improvement for any measures.

**Table 3:** PROMs completion rates at each time-point

	Intervention group				Control group			
Patient reported outcome measures, *n* (%)	T1	T2	T3	T4	T1	T2	T3	T4
EPIC	19 (66)	–	–	9 (31)	9 (75)	–	–	5 (42)
*PAM	26 (90)	18 (62)	15 (52)	9 (31)	11 (92)	–	–	10 (83)
*CASUN	21 (72)	17 (59)	10 (34)	12 (41)	9 (75)	–	–	10 (83)
EQ-5D	26 (90)	–	–	11 (38)	11 (92)	–	–	10 (83)
WEMBWS	27 (93)	–	–	12 (41)	11 (92)	–	–	10 (83)
EORTC	13 (45)	–	–	6 (21)	8 (67)	–	–	8 (67)

*T*, time-point, E*PIC* (Expanded Prostate Cancer Index Composite); *PAM* (Patient Activation Measure); *CASUN* (Cancer Survivors’ Unmet Needs); *EQ-5D* (The EuroQol five dimensions questionnaire); *WEMWBS* (Warwick-Edinburgh Mental Wellbeing Scale); *EORTC-QLQ* (European Organization for Research and Treatment of Cancer Quality of Life Questionnaire)

*Indicates PROMs assessed as part of interim analysis at T2 and T3 (intervention only)

**Table 4:** Descriptive PROM baseline, final and change data, stratified by group

	Intervention group			Control group		
	Pre-median (IQR)	Post-median (IQR)	Median change (IQR)	Pre-median (IQR)	Post-median (IQR)	Median Change (IQR)
EPIC (0–100) Higher score represents improvement	***N*** **=** 6			***N*** **=** 3		
Urinary summary	89.25 (82.00, 93.08)	87.89 (80.59–88.92)	− 2.08 (− 6.91, 2.08)	97.25 (84.04–98.63)	97.92 (71.88, 97.92)	− 2.08 (− 13.54, − 0.71)
Bowel summary	87.50 (82.14–92.86)	86.11 (82.14–92.86)	0.00 (− 3.57, 3.57)	94.64 (93.17, − 94.64)	100.00 (82.14, 100.00)	5.36 (− 11.029, 5.36)
Hormonal summary	87.05 (72.72–93.18)	87.50 (84.09–100.00)	− 1.14 (− 2.27, 15.91)	90.91 (72.72–95.45)	70.45 (70.45, 79.55)	− 2.27 (− 15.91, 6.82)
Sexual summary	9.62 (7.69–19.85)	17.31 (11.54–23.08)	3.85 (0.18, 11.54)	11.10 (10.36, 17.74)	9.60 (4.8, 20.10)	− 0.02 (− 5.56, 3.10)
PAM (0–100) Higher score represents improvement	***N*** **=** 8			***N*** **=** 10		
Score	69 (54.40, 75.00)	58 (52.25, 73.75)	2.95 (− 15.65, 7.70)	55.6 (55.60–63.10)	59.35 (53.20, 67.80)	0.00 (− 2.20, 4.70)
Level (1–4)	3.5 (2.50, 4.00)	3 (2.50, 4.00)	0.00 (− 1.00, 0.50)	3 (3.00–3.00)	3 (2.00–3.00)	0.00 (− 0.50,0.00)
CASUN Lower score represent improvement	***N*** **=** 8			*N* = 8		
Existential survivorship (0–14)	3.00 (0.00, 6.00)	1.5 (0.5–5.00)	0.00 (− 2.50, 1.00)	0.50 (0.00–1.00)	4.50 (0.00–8.50)	2.50 (0.00, 8.50)
Comprehensive cancer care (0–6)	1.00 (0.00–4.00)	4.00 (2.50–5.00)	2.00 (0.00, 3.50)	1.00 (0.50–4.50)	4.50 (1.00–5.00)	0.5 (− 0.50, 3.00)
Information (0–3)	1.00 (0.00–2.00)	1.00 (0.50–2.00)	0.00 (− 1.50–1.50)	0.00 (0.00–1.00)	1.00 (0.00–2.00)	0.00 (0.00, 1.00)
Quality of life (0–2)	0.00 (0.00–1.50)	1.50 (0.00–2.00)	0.00 (0.00–1.00)	0.00 (0.00, 0.50)	0.50 (0.00–1.00)	0.00 (−0.50,1.00)
Relationship (0–3)	0.50 (0.00–1.50)	0.50 (0.00–2.50)	0.00 (0.00–1.00)	0.00 (0.00, 0.50)	1.00 (0.00–1.50)	0.50 (0.00,1.50)
EQ-5D-5L Higher score represents improvement	*N* = 10			*N* = 10		
VAS (0–100)	68.00 (50.00–80.00)	60.00 (50.00–85.00)	0.00 (− 7.00, 5.00)	74.50 (60.00, 90.00)	83.00 (75.00–90.00)	2.50 (0.00, 30.00)
Utility index	0.80 (0.73, 0.86)	0.81 (0.75–0.86)	0.03 (− 0.06, 0.14)	0.84 (0.74, 0.85)	0.79 (0.73–1.00)	0.00 (0.00, 0.00)
WEMBWS (14–70) Higher score represents improvement	***N*** **=** 11			*N* = 10		
	50.00 (45.50–60.50)	55.00 (49.00–57.00)	− 1.00 (− 8.00, 10.00)	52.50 (43.00, 57.00)	52.00 (45.00–55.00)	2.50 (− 12.00, 6.00)

EORTC scores are not presented due to high levels of missing data

### Acceptability

The adapted TAM was returned by 11/29 participants. Results indicate the sHNA to be acceptable, with areas of refinement related to log on and password issues (Table 5). Only 2/5 practice nurses completed the measure, both indicating that they would be ‘somewhat willing’ to use the sHNA routinely.

**Table 5:** Patient acceptability of the technology

	Strongly disagree	Very much disagree	Disagree	Not sure	Agree	Very much agree	Strongly agree
Usefulness (*n*)							
I would expect CHAT-P to help me improve my care	0	0	0	3	7	1	0
I would expect CHAT-P to be useful to the doctors and nurses that care for me	0	0	0	1	10	0	0
I would expect CHAT-P to be useful in consultations	0	0	0	1	9	0	0
I expect using CHAT-P would help me to understand my condition	0	0	1	1	8	1	0
I expect CHAT-P to help me look after myself	0	0	0	4	5	2	0
Overall I expect CHAT-P to be of benefit to me	0	0	0	1	8	2	0
Ease of use (*n*)							
I found it easy to log on to CHAT-P	0	1	3	2	2	2	0
I found it easy to re-set my password when required	0	1	4	0	4	0	0
I found the screen format clear	0	0	0	0	9	1	0
I was able to identify what needed to be filled in quite easily	0	0	0	1	6	2	1
I found it straight forward to interact with CHAT-P	0	0	0	2	5	3	0
I think the presentation of CHAT-P is good and has a clear outline	0	0	1	2	6	1	0
I found the links to further information useful	0	0	0	3	5	2	0
Overall the system is easy to use	0	0	0	3	6	1	0

## Discussion

The primary aim of our feasibility study was to assess the recruitment, retention, acceptability, and engagement of patients and nurses with the sHNA.

Initial patient engagement with the sHNA was high, with all but one patient receiving nurse-led follow-up and an individualised care plan following the completion of the sHNA. The sHNA also proved useful in identifying concerning or ‘red flag’ symptoms for discussion, and helping practice nurses decide when to seek or arrange further medical care for the patients, thus contributing to important components of safety netting within primary care [17]. This coupled with positive feedback indicated by the TAM suggests that online assessments and joint care planning during nurse-led consultations are acceptable to men living with and beyond prostate cancer. However, care plans are often not routinely provided to cancer patients [18]. Furthermore, it is widely reported that older adults are underrepresented in cancer related-trials [19], yet ~ 50% of men recruited to this study were over the age of 76 years. Despite the positives that can be drawn from this, progression criteria in terms of recruitment and engagement were not met. Overall, patient recruitment was low for both the intervention group (22%) and control group (13%) and lower than the 61% recruitment of eligible men reported by a recent pilot study looking at a nurse-led psycho-education intervention for men with prostate cancer [20]. However, the mentioned study recruited only participants who self-reported unmet needs related to urinary, bowel, and sexual or hormone-related functioning/vitality. In contrast, our study included both a digital element and also a broader sample of men (i.e. those who had ever had a prostate cancer diagnosis, regardless of the perceived level of need). Whilst reasons for poor uptake were not recorded, a recent systematic review reported that retention is lower among cancer patients allocated to web-based intervention arms [21], and thus such interventions may also achieve lower recruitment. Difficulties engaging primary care practices to participate in the study and the mandatory Macmillan training for the nurses’ also impacted recruitment. Training dates were limited, and some practice nurses were not able to attend, and as such were not eligible to run an intervention site. Smaller than anticipated practice patient lists also limited the numbers of eligible participants.

Our descriptive analyses showed no difference between older and younger participant groups in terms of retention. However, the majority of men in the older age category received additional support and encouragement from a peer supporter ‘ITmate’ which likely enhanced their retention in the study. As such, the ITmate may represent a promising model for improving recruitment and retention rates among older adults for digital-based clinical trials. Furthermore, we observed that median time since diagnosis was longer among study completers (120 vs 60 months), but given the small number of participants other confounding factors e.g. co-morbidities and disease severity were not controlled for. As such, we make no inferences from this. However, our previously published qualitative study [22], concluded that the sHNA may be most relevant if implemented at an early stage closer to diagnosis. As such, time since diagnosis should be explored in a larger trial, with tailored strategies for retention developed if this factor is shown to be predictive of drop out.

Our findings demonstrate a potential for the sHNA to be implemented as intended, with ~ 70% of intervention participants accessing sHNA 1 and 2. However, sHNA completion rates failed to meet progression criteria, given the low number of participants who completed sHNA 3. This was due to a number of factors including a late starting site and cumulative effects of study withdrawals and death. Most patients opted for a telephone consultation rather than a face-to-face meeting with the nurse, suggesting this as an acceptable and likely time saving way to communicate. The median consultation length was 15 min. Duration included time taken by the nurses to open and log-in to the IT software, speak with the patient, and update their care plan and records. Previous studies evaluating specialist nurse-led telephone follow-up in men with prostate cancer have shown high levels of satisfaction, with patients finding the service both informative and useful [23]. Given the increasing role of primary care in cancer care follow-up, it is encouraging to see that telephone consultation remains a favourable way of contact; as when coupled with novel digital health technology as in our study, this has the potential to improve intervention accessibility and reach [24].

Practice nurses made seven patient referrals to other services. Whilst it is unclear from the findings if these referrals were above and beyond that of routine care, it does indicate a need for this type of follow-up. The vast majority of needs identified by patients through the sHNA were managed by the practice nurse, highlighting the ability of this professional group to deal with the broader concerns of men with prostate cancer and coordinate their care. Interventions using nurse navigators have previously been shown to improve patient experience and reduce problems in care for patients with breast, colorectal, and lung cancer [25]. Using the sHNA outcomes and patient clinical information, nurses were able to determine any problems requiring further management, and refer patients quickly and directly to a specialist nurse. Whilst this achieved coordination of care, integration in terms of a shared approach to patient management did not take place. The major limiting factor to the integration of care was a lack of integration between the IT platform that held the sHNA output and the NHS Trust clinical data management system. This necessitated a manual process for alerting specialist clinicians to study patients with forthcoming clinic appointments and required clinicians to register and login to a website outside of their clinical portal. As a result, and knowing practice nurses would be likely to make contact if required, clinicians failed to access the system.

Emotional concern was the most common domain accessed in the sHNA, suggesting that men seek greater support with emotional concerns. It is well evidenced that depression in men with prostate cancer is a significant and complex issue [26]; however, whilst this study was not powered to detect changes in outcomes, pre-and-post WEMWBS scores did not denote a change of improvement for the intervention. This suggests that sHNA and joint care-planning may not be enough to support the emotional needs of men with prostate cancer, but may help to identify men requiring emotional support.

The sHNA was perceived as useful by patients, with nurses’ indicating a willingness to implement it routinely. However, these findings should be interpreted with caution given the low patient numbers for the adjusted TAM. Acceptability of the study protocol was low when considering the high level of missing data within the PROMs. Reasons for missing data are not known, but may be indicative of the high volume of PROMs and resulting questionnaire fatigue. Further work is required to ensure questionnaires are suitable and acceptable to patients. This could include undertaking cognitive interviews with patients to ensure acceptability of the questionnaires [27], allowing for adaptions to be made where indicated, as well as user testing to ensure acceptability of the digital format they are presented in.

### Limitations

The biggest limitation of this study was its inability to engage general practices of sufficient size and lower than expected rates of recruitment. This likely impacted on the way in which the intervention was implemented as the sHNA was designed to help practice nurses embed cancer follow-up in the pathway of prostate cancer. Practice nurses are in a unique situation given their knowledge of the patient and their family to provide this additional follow-up support. However, research nurses were unable to replicate this patient relationship in practices that were not their own, likely impacting on recruitment rates and potentially patient experience. Furthermore, the extent to which the sHNA can be viewed as beneficial (e.g. contributing to early specialist referrals) beyond that of usual care is limited due to a lack of data collected from the comparative group. Finally, attrition rates were higher than expected, and poor completion of PROMs meant that limited efficacy testing of the intervention to impact on important factors associated with patient quality of life was not possible. As the study did not meet progression criteria in terms of recruitment, retention, or completion of outcome assessments, we felt it inappropriate to undertake a power calculation to determine a future study sample size. Instead, this study will be used to refine our implementation strategy, with the refined study design requiring further piloting prior to being tested in a fully powered RCT.

The current Quality Outcomes Framework (QOF) (performance management and payment of GPs in England) incentivises primary care to establish a cancer register and conduct a cancer care review within 6 months of diagnosis [23]. However, there remains an unstandardized approach to cancer follow-up. A lack of remuneration, rather than a lack of interest, may explain the encountered difficulties with engaging practices in the study, along with concerns recorded anecdotally regarding the time required to implement the intervention.

## Conclusion

In conclusion, although the findings from this feasibility study failed to meet the progression criteria for undertaking a randomised controlled trial in the current context of primary care, the sHNA was acceptable to patients and showed promise in facilitating focused and timely patient consultations, identifying ‘red flag’ symptoms, and assisting with coordinated referrals to more specialist care. Furthermore, practice nurses were open to implementing the sHNA into their routine practice, but there may be a need for practices to be financially incentivised to take on this activity. The findings of this study underline known challenges to implementing digital technology to facilitate care integration across healthcare services. Whilst our study showed promise for improving care coordination, full integration did not occur as intended, and further work is required to overcome barriers related to IT system integration. However, with growing numbers of survivors, the landscape of cancer is changing [28], and primary care and specialist care communication is essential to ensuring the long-term and optimal management of these patients. With some small refinements to the sHNA together with IT systems in place that can support integration across primary and secondary care, there is clear potential for this system to facilitate holistic integrated care.
